# Book Reviews

**Published:** 1896-05

**Authors:** 


THE AMERICAN YEAR-BOOK OF MEDICINE AND SURGERY. Being a Yearly Digest of Scientific Progress and Authoritative Opinion in all Branches of Medicine and Surgery, drawn from Journals, Monographs and Text-Books of the leading American and Foreign Authors and Investigators. Collected and arranged with Critical Editorial Comments. By J. M. Baldy, M. D., C. H. Burnett, M. D., Archibald Church, M. D., C. F. Clarke, M. D., J. Chalmers DaCosta, M. D., W. A. N. Dorland, M. D., V. P. Gibney, M. D., Homer W. Gibney, M. D., Henry A. Griffin, M. D., John Guitéras, M. D., C. A. Hamann, M. D., H. F. Hansell, M. D., W. A. Hardaway, M. D., T. M. Hardie, B. A., M. B., C. F. Hersman, M. D., B. C. Hirst, M. D., E. Fletcher Ingals, M. D., W. W. Keen, M. D., H. Leffman, M. D., V. H. Norrie, M. D., H. J. Patrick, M. D., William Pepper, M. D., D. Riesman, M. D., Louis Starr, M. D., Alfred Stengel, M. D., G. N. Stewart, M. D., and Thompson S. Westcott, M. D. Under the general editorial charge of GEORGE M. GOULD, M. D. Profusely illustrated with numerous woodcuts in text and thirty-three half-tone and colored plates. Royal 8vo, pp. vi.—1183. Price, $6.50. Philadelphia: W. B. Saunders. 1896.

This latest addition to the series of year-books is the most colossal of its group. There seems to be a desire on the part of each of the American medical publishers to issue an annual epitomé of the progress of medical science, which for convenience is called a year-book; but this one, it must be confessed, overreaches all the others in size and in general comprehensiveness. Whether it will become more popular with the profession remains to be seen.

Of the twenty-eight editors, including the editor-in-chief, sixteen of them reside in Philadelphia. While this may be advantageous to the editor in charge in facilitating proof-reading and in otherwise hastening the publication of the work, it would seem that to distribute the work over a wider area of country would give it greater national coloring and importance.

Some idea of the magnitude of the treatise may be obtained from the fact that general medicine occupies 162 pages of the book; general surgery, 180 pages; obstetrics and gynecology, 182 pages; pediatrics, 62 pages; nervous and mental diseases, 79 pages; dermatology and syphilis, 69 pages; orthopedic surgery, 7 pages; ophthalmology, otology, rhinology and laryngology, 283 pages; pathology, 99 pages; materia medica, experimental therapeutics and pharmacology, 52 pages; anatomy and physiology, hygiene and sanitary chemistry, medical jurisprudence and clinical chemistry, 28 pages. In the last section, page 1123, we notice a statement to the effect that the office of coroner in the state of New York has been abolished. This is an error, more's the pity; but if the last legislature had performed its full duty under the constitution the office would have been abolished.

An elaborate index, of fifty-five three-column pages, furnishes a fitting and adequate key to this excellent grouping of the salient points in medical progress for a year.

---

FUNCTIONAL AND ORGANIC DISEASES OF THE STOMACH. By SIDNEY MARTIN, M.D., F.R.S., F.R.C.P., Assistant Physician and Assistant Professor of Clinical Medicine at University College Hospital; Assistant Physician to the Hospital for Consumption and Diseases of the Chest, Brompton. Octavo, pp. xvi.—505. With fifty-seven illustrations. Edinburgh and London: Young J. Pentland. Philadelphia: J. B. Lippincott Co. 1895.

This treatise will be welcomed by English-speaking people, as it is many years since diseases of the stomach have been discussed in a systematic treatise. The author begins with a chapter on the anatomy and physiology of the stomach, of which little need be said beyond commending it as a careful exposition of the subject. In the next chapter he discusses the digestibility of various articles of diet. This involves cookery and other methods of preparing food. A table of the digestibility of articles of diet in the stomach affords an interesting study. Some of the information to be gleaned from it will be found in opposition to popular notions.

In the next two chapters the author presents an able discussion of the pathology of the indigestion of food. A careful study of these chapters by every physician engaged in general practice is recommended. Methods of examination of the functions of the stomach in disease form the subject of Chapter 5. In it the author describes simple methods which he asserts can be performed, to a large extent, with as much ease as is the examination of the urine. The author takes up the symptoms referable to indigestion of food in Chapter 6, and in Chapter 7 discusses functional disorders of the stomach. In the latter chapter will be found an analysis of gastric irritation, caused by neuroses of the stomach, which will prove of much interest to the reader. Mechanical and active congestion of the stomach, gastritis and gastric catarrh are dealt with in Chapter 8, the latter in a most clear and satisfactory manner. Toxic and infective gastritis, together with atrophy and degeneration of the stomach, form the subject matter of Chapter 9.

In the next three chapters the author considers the treatment of acute and chronic affections of the stomach. In Chapter 10 he presents their medicinal treatment; in Chapter 11 he offers their treatment by diet and discusses the value of the different prepared foods—peptonised, malted, fatty and the like; while in Chapter 12 their general hygienic treatment is set forth. In this chapter the value of lavage is properly estimated. Martin says it is of great benefit in certain cases, but that it is a method that has been much abused and must be applied with great circumspection.

The next chapter, 13, is set apart to the consideration of bleeding from the stomach, and in Chapter 14 dilatation of the stomach is fully discussed. Ulcer of the stomach is allotted to Chapter 15, which is a most interesting one and deserves careful reading. Finally, cancer of the stomach is dealt with in Chapter 16 in a masterly manner.

From this epitomé may be gathered the scope and import of the treatise. It is written with conciseness and in the best of English. The author shows he is master of the subject in every chapter of the book. It is well printed and has some excellent illustrations.

---

An American Text-Book of Surgery, for Practitioners and Students. By Charles H. Burnett, M. D., Phineas S. Conner, M. D., Frederic S. Dennis, M. D., William W. Keen, M. D., Charles B. Nancrede, M. D., Roswell Park, M. D., Lewis S. Pilcher, M. D., Nicholas Senn, M. D., Francis J. Shepherd, M. D., Lewis A. Stimson, M. D., William Thompson, M. D., J. Collins Warren, M. D., and J. William White, M. D. Edited by William W. Keen, M. D., LL.D., and J. William White, M. D., Ph.D. Second edition, carefully revised. Illustrated. Imperial 8vo, pp. xiv.—1248. Price, $7.00 cloth; $8.00 sheep and $9.00 half-Russia. Philadelphia: W. B. Saunders, 925 Walnut street. 1895.

The first edition of this work appeared in the Autumn of 1892, and it rapidly crept into favor with the profession. It has already been adopted as a text-book by over sixty medical schools in America and has been accorded a large foreign sale. It is not surprising, therefore, that the first edition was early exhausted and that a second is already meeting with large demand.

The editors have taken advantage of this opportunity to incorporate some of the many advances surgery has made during the past three years in this edition. The more notable of these relate to intracranial and spinal surgery; surgery of the chest; surgery of the digestive tract; intestinal anastomosis with the Murphy button, and a chapter on symphyseotomy. Additions have also been made to the sections relating to fractures and dislocations, appendicitis, hernia and amputations of the breast.

In a former review we referred to the excellent character of its illustrations. In the present edition many of these have been redrawn and a number of new ones have been added. There are a number of admirable features about this work and we have no doubt it will continue to hold a popular place in the literature of surgery. The mechanical execution is all that could be desired, but we advise intending purchasers to secure it bound in leather, as its weight is too great to be sustained long by cloth.

The tendency of the present is towards the encyclopedic form of literature, and if all treatises written on this plan were as free from defects as this American text-book of surgery, it would go far toward the justification of this method. This book is especially adapted to the wants of the physician who must limit his library to a few well-selected books.

---

Twentieth Century Practice. An International Encyclopedia of Modern Medical Science. By leading authorities of Europe and America. Edited by Thomas L. Stedman, M. D., New York City. In twenty volumes. Volume VI.: Diseases of the Respiratory Organs. New York: William Wood & Co. 1895.

Owing to delay in the receipt of manuscript, the sixth volume of this interesting series appears before the fifth. It is devoted to the consideration of diseases of the respiratory tract, and very properly begins with those of the nose. This section is written by Proctor James, of London, and contains the essential details commensurate with our present knowledge of the subject. Following this, diseases of the accessory sinuses of the nose are taken up by Jonathan Wright, of Brooklyn. The antrum receives due consideration. It is an important locality and possesses great anatomical and pathological interest.

Next come diseases of the nasal pharynx and pharynx, to which E. J. Moure, of Bordeaux, devotes over 100 pages. Syphilitic and tubercular diseases of this region are always of great importance and are intelligently dealt with by this author. Moure also treats of diseases of the tonsils in a section of about fifty pages, which is one of the most important in the book. Neglected disease of these organs often lead to serious or incurable results. Every physician should pay close attention to this subject. A section is devoted to diseases of the ear, written by Albert H. Buck, of New York. This author is experienced and always interesting; never more so, however, than in these pages.

The section devoted to diseases of the larynx is written by Francke H. Bosworth, of New York, who is amply qualified to deal with this important subject. That its gravity is appreciated may be inferred from the fact that he devotes 168 pages to its consideration, much of which treats of syphilitic and tubercular affections of this organ. Foreign bodies in the larynx and the laryngeal diseases of childhood, always fraught with intense interest, are here amply set forth.

In the next section we find diseases of the trachea and bronchial tubes dealt with under the joint authorship of Thomas Grainger Stewart and George Alexander Gibson, of Edinburgh. In the 186 pages devoted to this branch of the subject will be found its latest exposition in the most concise manner.

Not of the least importance is the last section of the book, in which are considered diseases of the lungs (excluding croupous pneumonia and tuberculosis) by Winslow Anderson, of San Francisco, a subject this author is abundantly able to deal with. The two affections which he omits will be considered in a later volume, together with the other infectious diseases.

Taken altogether, this volume may be fairly estimated to be the most important of the series thus far published.

Practical Urinalysis and Urinary Diagnosis. A Manual for the Use of Physicians, Surgeons and Students. By Charles W. Purdy, M. D., Queen's University; Fellow of the Royal College of Physicians and Surgeons, Kingston; Professor of Urology and Urinary Diagnosis at the Chicago Post-Graduate Medical School. Second revised edition. With numerous illustrations, including photo-engravings and colored plates. In one crown 8vo volume, 360 pages. In extra cloth, $2.50 net. Philadelphia: The F. A. Davis Co., Publishers, 1914 and 1916 Cherry street. 1895.

It is just a year ago that we wrote a notice of the first edition of this book. That a second edition is demanded so soon is sufficient testimony to the popularity of the work. There is very little change made in this edition, excepting to correct errors and to add a few plates, hence there is very little to say in regard to it beyond calling attention to its renewed publication.

The following from our first notice is, however, pertinent to reproduce: an appendix is added to the book, which treats of the examination of urine for life insurance. Every examiner should familiarise himself with this important branch of the subject. Life insurance companies might, with great propriety, reprint this section and distribute it among their examiners. A good index closes this well-printed, interesting and valuable treatise, which we commend as one of the best of its kind.

A Guide to the Practical Examination of Urine, for the Use of Physicians and Students. By James Tyson, M.D., Professor of Clinical Medicine in the University of Pennsylvania, and Physician to the Hospital of the University, etc. Duodecimo, pp. xii.—276. Ninth edition, revised and corrected. With a colored plate and wood engravings. Price, $1.25. Philadelphia: P. Blakiston, Son & Co., 1012 Walnut street. 1895.

No similar treatise has been so long before the professional public and none comes so near to being a classic on the subject of which it treats as does this one. No large additions have been made since a former edition appeared, but some minor paragraphs have been omitted.

A French translation of this useful book has lately appeared in Paris, under the editorship of Drs. Gautrelet and A. S. Clarke, and published by the Société d'Éditions Scientifiques.

---

Surgery. A Practical Treatise, with Special Reference to Treatment. By C. W. Mansell Moullin, M. A., M. D., Oxon.; Fellow of the Royal College of Surgeons; Surgeon and Lecturer on Physiology to the London Hospital, etc.; assisted by various writers on special subjects. With 623 illustrations. Royal 8vo, pp. 1250. Third American edition, revised and edited by John B. Hamilton, M. D., LL.D., Professor of the Principles of Surgery and Clinical Surgery, Rush Medical College, Chicago; Professor of Surgery, Chicago Polyclinic; Surgeon, formerly Supervising Surgeon-General, U. S. Marine Hospital Service, etc., etc. Price, $6.00. Philadelphia: P. Blakiston, Son & Co., 1012 Walnut street. 1895.

The first edition of this work received appropriate notice in these columns, in which it was considered in some detail. We did not receive the second edition, which was edited by Gen. Hamilton, as is also the present one.

The appearance of successive editions, with but two years intervening between each, is indicative of the firm hold it has taken upon surgeons; this, too, in spite of the fact that numerous other surgical treatises have appeared since this one first issued. The author is fortunate in the selection of his American editor, who well knows surgeons and their needs in this country, and whose literary attainments specially fit him for the task he has assumed.

Even though but two years have elapsed since the last edition was issued, it has yet been found necessary to make considerable revisions of certain parts of the work, particularly those relating to intracranial and spinal surgery and intestinal anastomosis. The method devised and so skilfully practised by Professor Murphy, of Chicago, is described with much precision and clearness.

In the description of the surgery of fibroid tumors of the uterus, Professor Hamilton gives preference to the elastic ligature of Pozzi over the Koeberlé serre-neud. Vaginal hysterectomy, after Péan, is described, but no opinion is expressed as to its relative merits.

The additions and comments made by the editor are inclosed in brackets, which makes identification easy. Taken altogether, this is a treatise that no modern surgeon can afford to do without.

---

TRANSACTIONS OF THE AMERICAN GYNECOLOGICAL SOCIETY. Vol. XX., for the year 1895. Edited by HENRY CLARK COE, M.D., Secretary, New York. Published by the society. Philadelphia: William J. Dornan, Printer. 1895.

This volume is the most elaborate of any yet published by this society, containing 633 pages. The papers, thirty-one in number, cover almost the entire field of operative gynecology, and they are discussed with the ability that characterises the work of this society. The paper by Dr. Charles Jacobs, of Brussels, on vaginal hysterectomy, to which he has given the somewhat peculiar title of Indications for total castration of the vagina, will naturally attract considerable attention. He gives the most recent statistics of this operation as follows: Landau, 141 cases, 2 deaths; Léopold, 44 cases, 1 death; Sænger, 17 cases, 2 deaths; Péan, 450 cases, 12 deaths; Richelot, 219 cases, 11 deaths; Doyen, 253 cases, 18 deaths; Segmond, 200 cases, 14 deaths; Gallet, 29 cases, 2 deaths; Jacobs, 403 cases, 12 deaths. Total, 1,756 cases, 74 deaths; 4.2 per cent. mortality. The volume closes with a memorial of Dr. William Goodell, to which is appended a list of the papers published by the deceased, 113 in all, giving the date and place of publication. To this volume is appended a general index of the twenty volumes published by the society, which involved a great deal of hard work, but amply repays for it in the convenience it serves.

Materia Medica and Therapeutics. A Practical Treatise, with Especial Reference to the Clinical Application of Drugs. By John V. Shoemaker, A.M., M. D., LL. D., Professor of Materia Medica, Pharmacology, Therapeutics and Clinical Medicine, and Clinical Professor of Diseases of the Skin, in the Medico-Chirurgical College of Philadelphia; Physician to the Medico-Chirurgical Hospital, Philadelphia, etc., etc. Third edition, thoroughly revised. Reset with new type and printed from new electrotype plates. Royal 8vo, pp. ix.—1108. Extra cloth, $5.00 net; sheep, $5.75 net. Philadelphia: The F. A. Davis Co., Publishers, 1914 and 1916 Cherry street. 1895.

The third edition of this work comes to us with the two volumes of former editions combined into one. This improvement is in the interest of convenience and will be appreciated by the many readers of the treatise.

This edition presents whatever has transpired during the two past years, either in the chemical laboratory or in the clinic room, that may pertain to materia medica and therapeutics. This has involved the publication of much additional matter, especially in the descriptions of the coal-tar series. Treatment by means of animal extracts and immunised serum or antitoxins has received much attention, in an endeavor to bring the subject forward to our present state of knowledge.

In the enumeration of preparations, the author conforms to the United States Pharmacopeia of 1890, and he has thoroughly revised the indexes. A consideration of agencies other than medicine, in their application to the treatment of disease, forms an interesting and useful section, which is comprised in part third of the treatise. Those who possess the former editions will eagerly seek this one and those who do not will profit in its reading.

---

A Manual of Operative Surgery. By Lewis A. Stimson, B. A., M. D., Professor of Clinical Surgery in the University of the City of New York. New (third) edition. In one royal 12mo volume of 614 pages, with 306 illustrations. Cloth, $3.75. Philadelphia: Lea Brothers & Co., Publishers. 1895.

This book is familiar to the profession through the two editions that have already been exhausted, hence there is little to be said in regard to it in addition to what we have heretofore written in noticing the first and second editions. The author, who is well known throughout the country as a progressive surgeon, has utilised this opportunity to bring his work forward and place it abreast of present conditions. He says in order to do this it has been found necessary almost wholly to rewrite the book. The principal changes, however, will be found in the chapters relating to surgery of the cranium and abdomen. One of the advantages of this book is its convenient size, and a busy surgeon will often avail himself of the opportunity to consult it on that account. It is one of the best surgical manuals extant.

---

PRINCIPLES OF SURGERY. By N. SENN, M. D., Ph. D., LL. D., Professor of Practice of Surgery and Clinical Surgery in Rush Medical College, Chicago; Professor of Surgery in Chicago Polyclinic; Attending Surgeon to the Presbyterian Hospital; Surgeon-in-Chief to St. Joseph's Hospital; Ex-President American Surgical Association, etc., etc. Second edition, thoroughly revised. Illustrated with 178 wood-engravings and five colored plates. Royal octavo, pp. xvi.—656. Extra cloth, $4.50 net; sheep or half-Russia, $5.50 net. Philadelphia: The F. A. Davis Co., Publishers, 1914 and 1916 Cherry street.

In our notice of the first edition of this treatise we remarked that it was based largely on the personal experience and study of the author. This feature is of much advantage where an author possesses so much skill in observing facts and accuracy in recording them as does Senn. The five years that have elapsed since the appearance of the first edition have been fruitful in pathological advances and have enriched the whole surgical field with many valuable additions. Seizing upon this opportunity, this author has brought his treatise forward, thus making it conform to present conditions. New material has been added and new illustrations have been supplied.

It must be remembered that this is a treatise on the principles of surgery, and is not intended to embrace the whole field of surgical science and art. He has skilfully kept within his original purpose, confining his treatise to the application of surgical principles to certain pathological conditions. He makes a prominence of the relation of bacteriology to etiology and pathology; he discusses the cellular process of regeneration and inflammation and does not forget to give due prominence to diagnosis, prognosis and treatment.

It is the most classic of the products from the prolific pen of this distinguished author.

